# Daylight photodynamic therapy versus cryosurgery for the treatment and prophylaxis of actinic keratoses of the face − protocol of a multicenter, prospective, randomized, controlled, two-armed study

**DOI:** 10.1186/s12895-017-0064-7

**Published:** 2017-10-25

**Authors:** E. Kohl, M. Koller, F. Zeman, R.-M. Szeimies, W. G. Philipp-Dormston, W. Prager, P. A. Gerber, S. Karrer

**Affiliations:** 1 Department of Dermatology, University Hospital Regensburg, 93042 Regensburg, Germany; 2 Center for Clinical Studies, University Hospital Regensburg, 93042 Regensburg, Germany; 3 Department of Dermatology and Allergology, Vest Hospital, Academic Teaching Hospital University of Bochum, 45657 Recklinghausen, Germany; 4 Hautzentrum Köln, 50966 Köln, Germany; 5 Prager & Partner, 22609 Hamburg, Germany; 6 Department of Dermatology, Medical Faculty, Heinrich-Heine University, 40225 Düsseldorf, Germany; 7 Department of Dermatology, University Hospital Regensburg, Franz-Josef-Strauss-Allee 11, 93053 Regensburg, Germany

**Keywords:** PDT, Study protocol, Cryosurgery, Methyl aminolevulinate, Daylight, AK, Skin aging, Prevention, Photorejuvenation

## Abstract

**Background:**

Photodynamic therapy with daylight (DL-PDT) is efficacious in treating actinic keratosis (AK), but the efficacy of field-directed, repetitive DL-PDT for the treatment and prophylaxis of AK in photodamaged facial skin has not yet been investigated.

**Methods/design:**

In this multicenter, prospective, randomized, controlled, two-armed, observer-blinded trial, patients with a minimum of 5 mild-to-moderate AK lesions on photodamaged facial skin are randomly allocated to two treatment groups: DL-PDT with methyl aminolevulinate (MAL) and cryosurgery. In the DL-PDT group (experimental group), 5 treatments of the entire face are conducted over the course of 18 months. After preparation of the lesion and within 30 min after MAL application, patients expose themselves to daylight for 2 h. In the control group, lesion-directed cryosurgery is conducted at the first visit and, in the case of uncleared or new AK lesions, also at visits 2 to 5. The efficacy of the treatment is evaluated at visits 2 to 6 by documenting all existing and new AK lesions in the face. Cosmetic results and improvement of photoaging parameters are evaluated by means of a modified Dover scale.

Primary outcome parameter is the cumulative number of AK lesions observed between visits 2 and 6.

Secondary outcome parameters are complete clearance of AK, new AK lesions since the previous visit, cosmetic results independently evaluated by both patient and physician, patient-reported pain (visual analogue scale), patient and physician satisfaction scores with cosmetic results, and patient-reported quality of life (Dermatology Life Quality Index). Safety parameters are also documented (adverse events and serious adverse events).

**Discussion:**

This clinical trial will assess the efficacy of repetitive DL-PDT in preventing AK and investigate possible rejuvenating effects of this treatment. (Trial registration: ClinicalTrials.gov Identifier: NCT02736760).

**Trial registration:**

ClinicalTrials.gov Identifier: NCT02736760. Study Code Daylight_01. EudraCT 2014–005121-13.

## Background

Actinic keratosis (AK) is one of the most frequently diagnosed skin diseases in dermatological practice. Clinical examination of the study participants of the Rotterdam cohort study aged >45 years showed a prevalence of up to 49% in men and 28% in women [1, 2]. Therefore, treatment of AK causes high costs for health insurances [3]. Consequent treatment of AK is necessary because of the possibility of malignant transformation. AK lesions are regarded as in situ squamous cell carcinomas [4] that progress to invasive squamous cell carcinomas in up to 20% of patients [5–7]. In 2015, ‘multiple actinic keratoses’ defined as more than 5 AK lesions per year or field cancerization of >4 cm^2^ have been acknowledged as a new occupational disease in Germany [8]. National and international guidelines recommend the treatment of AK upon diagnosis to prevent further progression into squamous cell carcinoma [9].

Besides several other treatment options, photodynamic therapy (PDT) with red light for illumination is characterized by high efficacy and good esthetic results, largely independent of patient adherence. Daylight-PDT (DL-PDT) is a new treatment approach that uses natural sunlight for illumination. DL-PDT is associated with less discomfort and pain compared to standard treatment protocols, and its efficacy in treating AK seems to be equal to that of conventional PDT with red light [10–12].

However, clinical trials investigating the efficacy of field-directed, repetitive DL-PDT for the prophylaxis of AK are lacking [11, 13, 14] as well as knowledge about the esthetic and rejuvenating effects of repetitive DL-PDT.

Therefore, the present study investigates the prophylactic effect of repetitive DL-PDT on the occurrence of AK as compared to lesion-directed cryosurgery. In addition, the rejuvenating effects of DL-PDT with regard to the clinical signs of photoaged skin are evaluated.

## Methods/design

### Study design

This study was designed as a multicenter, prospective, randomized, controlled, two-armed study that compares two different treatments. MAL application followed by illumination with daylight (DL-PDT) represents the experimental treatment, and cryosurgery as a standard lesion-directed therapy of AK is investigated as a reference therapy. Patients are randomly allocated to one of the two treatment methods.

The study was approved by the Institutional Review Board and the Ethics Committee of the University of Regensburg (Approval number: 15–112-0068) as well as by the German Federal Institute for Drugs and Medical Devices (Approval number: 61–3910-4,040,415). The study was registered at EU Clinical Trials Register (EudraCT Number: 2014–005121-13) on November the 17th 2014 and at clinicaltrials.gov (ClinicalTrials.gov Identifier: NCT02736760) in March 2016. The clinical trial was not registered retrospectively. The SPIRIT guidelines were followed in this study protocol.

Written informed consent will be obtained from each patient before enrolment. This is an ongoing clinical trial.

### Inclusion and exclusion criteria

**Fig. 1:**
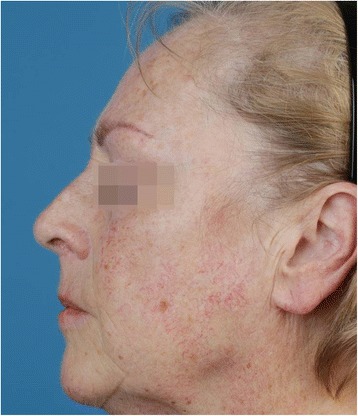
Potential study patient. Face of a potential study participant with Glogau Photodamage Classification Type III (advanced). Multiple visible AK, sallow skin discoloration with teleangiectasias and wrinkles

The main inclusion criteria are clinical diagnosis of actinic keratosis (AK), a minimum of 5 non-hyperkeratotic, non-pigmented AK lesions in the face (Olsen grading I or II), Glogau Photodamage Classification Type II (moderate) to IV (severe), Fitzpatrick skin type I to IV, age ≥ 40 years, a negative pregnancy test in women of child-bearing age, and written informed consent (Fig. 1).

The main exclusion criteria are known intolerance or allergy to MAL or to any other ingredient of Metvix® 160 mg/g cream or Actinica® lotion (both Galderma Laboratorium GmbH, Düsseldorf, Germany), photosensitivity, diagnosis of porphyria, hyperkeratotic or pigmented AK in the face, malignant skin tumors in the face or on the scalp, clinically relevant suppression of the immune system, pregnancy or lactation, medical history of PDT in the face during 6 months preceding study treatment, rejuvenating treatment of the face 3 months preceding study treatment or planned esthetic treatments in the face in the next 24 months, other topical treatments of AK in the face during 4 weeks preceding study treatment or systemic treatment with retinoids, and suspected lack of compliance (e.g. dementia).

### Study treatment

At every visit, the location of each AK lesion is documented within a chart and additionally marked on plastic sheets. The plastic sheets are marked with reference points of the face of each study participant, ensuring that the lesions will be found again at the next visit. Photo-documentation is carried out in a standardized manner at each visit.

Photodamage variables to be evaluated are fine lines, mottled pigmentation, skin color, tactile roughness, sallowness, teleangiectasias, deep wrinkles, facial erythema, sebaceous gland hyperplasia, and the global score for photoaging. Each photodamage variable is recorded on a 5-point scale (0–4, higher points indicating higher damage, modified after a scale from Dover et al. and Zane et al.) (Table 1) [15, 16].

**Table 1:** Photodamage Score

Score	0	1	2	3	4
Global score for photoaging	facial skin smooth to the touch, no significant fine lines, uneven pigmentation, deep wrinkles, erythema, teleangiectasias, sebaceous gland hyperplasia on the cheeks, forehead, or in the perioral area	one area (cheeks, forehead, or perioral area) of significant roughness, deep wrinkles, erythema, teleangiectasias, sebaceous gland hyperplasia, dyspigmentation or fine lines	two areas of significant roughness, deep wrinkles, erythema, teleangiectasias, sebaceous gland hyperplasia, dyspigmentation or fine lines or one area of significant roughness, deep wrinkles, erythema, teleangiectasias, sebaceous gland hyperplasia, dyspigmentation and fine lines	three areas of significant roughness, deep wrinkles, erythema, teleangiectasias, sebaceous gland hyperplasia, dyspigmentation or fine lines or one area of significant roughness, deep wrinkles, erythema, teleangiectasias, sebaceous gland hyperplasia, dyspigmentation and fine lines	facial skin with photodamage higher than grade 3
Fine lines	none	very few, widely disseminated lines	some discreet lines	a moderate number of lines, in close proximity	many lines, in close proximity
Mottled pigmentation	evenly pigmented skin	minor hypo-pigmentation or hyper-pigmentation	small areas of very light hypo-pigmentation or hyper-pigmentation or moderately sized areas of light hypo-pigmentation or hyper-pigmentation	moderate hypo-pigmentation or hyper-pigmentation or small areas of pronounced hypo- pigmentation or hyper-pigmentation	major hypo- pigmentation or hyper-pigmentation
Skin color	pale pink	pale	yellowish, greyish	pale, slightly yellowish-greyish	pale, distinctly yellowish-greyish
Tactile roughness	soft skin	soft, some rough areas	minor roughness	moderate roughness	major roughness
Teleangiectasias	none	rare, widely disseminated	some discreet teleangiectasias	a moderate number of teleangiectasias, in close proximity	many teleangiectasias, in close proximity
Deep wrinkles	none	superficial wrinkles in one area	superficial wrinkles in more than one area or moderately deep wrinkles in one area	moderately deep wrinkles in more than one area or deep wrinkles in one area	deep wrinkles in more than one area
Facial erythema	none	small areas with slight erythema	small areas with moderate erythema or moderately sized areas with slight erythema	moderately sized areas with erythema	large areas with erythema
Sebaceous gland hyperplasia	none	some in one area	some in several areas or several in one area	a moderate number in several areas or many in close proximity in one area	many in close proximity in more than one area

Photodamage variables to be evaluated are fine lines, mottled pigmentation, tactile roughness, sallowness, teleangiectasias, and global score for photoaging. Each photodamage variable is recorded on a 5-point scale (0–4, higher points indicating higher damage, modified after Dover 2005 und Zane 2007)

**Table 2:** Study flow chart

Time	- 4 weeks – 0 d+	d 0+	3 months after V1^#^	3 months after V2^#^	6 months after V3^#^	6 months after V4^#^	6 months after V5^#^
Visit	Screening	1	2	3	4	5	6
Study treatment Test-group: Fullface-Daylight-PDT		1.PDT	2.PDT	3.PDT	4.PDT	5.PDT	
Study treatment Control-group: cryosurgery		Cryo	Cryo°	Cryo°	Cryo°	Cryo°	
Documentation of AK lesions in the face		X	X	X	X	X	X
Randomization	X						
Obtaining Informed Consent	X						
Check of Inclusion/ Exclusion Criteria	X	X	X	X	X	X	X
Medical history	X						
Photodocumentation		X	X	X	X	X	X
Assessment of photoaging parameters		X	X	X	X	X	X
Documentation of newly occurred malignant skin tumors in the face		X	X	X	X	X	X
Documentation of AEs and SAEs		X	X	X	X	X	X
Pain score (VAS) during treatment		X	X	X	X	X	
Skin-related quality of Life (DLQI)	X						X
Patient satisfaction with cosmetic results			X	X	X	X	X
Investigator satisfaction with cosmetic results			X	X	X	X	X
Local skin reactions		X	X	X	X	X	
Pregnancy test	X	X	X	X	X	X	

+: Screening and Visit 1 may take place on the same day

# +/− 4 weeks

Cryo: Cryosurgery

° cryosurgery in the case of uncleared or newly developed AK lesions

Patient and investigator satisfaction with the cosmetic outcome is evaluated from visit 2 onwards according to the following scale: very satisfied, moderately satisfied, slightly satisfied and not satisfied.

Pain during treatment is assessed by means of a visual 11-point scale (VAS-scale, 0, none, to 10, insufferable pain). The patients mark their level of pain on the VAS. The investigator rates and documents erythema and edema after illumination with daylight or cryotherapy by means of a 5-point scale: 0 (no), 1 (mild), 2 (moderate), 3 (marked), and 4 (severe). From the second visit onwards, patients are asked if they have experienced any side effects or any adverse events. (Table 2)

#### MAL application followed by illumination with daylight

Each patient randomized to the PDT arm of this trial receives 5 treatments with daylight PDT. For each PDT session, an organic sunscreen (Actinica® lotion, LSF 50+, Galderma Laboratorium, Düsseldorf, Germany) without any mineral filters is applied onto the entire face and other parts of the body that will be exposed to daylight. After an absorption time of at least 15 min, the surface of the AK lesions is gently prepared with a curette or a scalpel to remove scales and crusts and to roughen the lesions’ surface. Then, a thin layer of MAL cream (Metvix®, Galderma Laboratorium, Düsseldorf, Germany) is uniformly applied onto the entire face including the hairless forehead, but sparing the eyelids, lips, and ears. Covering the entire face with a thin layer using a spatula approximately requires 1 tube (2 g) of MAL cream. No occlusive dressing is conducted. Within 30 min after MAL application, patients expose themselves to daylight for 2 h. After daylight exposure, patients return to the clinic or practice and are instructed in washing off the MAL cream with clear water. This treatment regimen is provided at visit 1 and repeated after 3 months, 6 months, 12 months, and 18 months (visits 1 to 5). DL-PDT can only be carried out from March until October, in non-rainy weather, and an outdoor temperature of at least 10 °C.

#### Cryosurgery

In the control-group, one single freeze–thaw cryosurgery is conducted at visit 1, using an open spraying procedure with liquid nitrogen for each AK lesion. After formation of an ice ball, freeze time starts that should be between 5 and 10 s per lesion. At visits 2 to 6, the efficacy of the treatment is evaluated by the investigator. Cryosurgery will also be conducted at visits 2 to 5 in the case of non-cleared or newly developed AK lesions.

### Outcome assessment

The primary outcome parameter is the cumulative number of AK lesions observed between visits 2 and 6.

Secondary outcome parameters are complete clearance of AK on patient and lesion basis, new AK lesions since the last visit, cosmetic results as evaluated on assessment scales, patient-reported pain (visual analogue scale, VAS), local reactions, patient satisfaction score with cosmetic results, physician satisfaction score with cosmetic results, and patient-reported quality of life (Dermatology Life Quality Index, DLQI).

In addition, safety parameters are documented (adverse events and serious adverse events, AE and SAE).

Complete clearance is documented if a lesion is no longer visible and also imperceptible to the touch [17]. Photodamage variables to be evaluated are fine lines, mottled pigmentation, skin color, tactile roughness, sallowness, teleangiectasias, deep wrinkles, facial erythema, sebaceous gland hyperplasia, and global score for photoaging. Each photodamage variable is recorded on a 5-point scale (0–4, higher points indicating higher damage, modified after Dover and Zane) [15, 16].

Patient and physician satisfaction with esthetic outcome is assessed at visit 2 to 6. Patients and physicians can choose from very satisfied, satisfied, moderately satisfied, and not satisfied.

### Statistical considerations

#### Study hypothesis

The study objective can be formulated as a test of the null hypothesis H0: μ1 = μ0 versus the alternative hypothesis H1: μ1 ≠ μ0, where μ1 and μ0 represent the mean cumulative number of AKs in the MAL/daylight-PDT and cryosurgery group.

#### Sample size

Sample size calculation is based on the primary endpoint, that is the cumulative number of AK lesions. According to clinical experience, we expect a mean cumulative number of 10 AK lesions in the daylight-PDT group versus 14 lesions in the cryosurgery group with a standard deviation of 5 in both groups. To detect a difference of 4 with a standard deviation of 5 with a power of 1 − β = 80% at a two-sided alpha significance level of 0.05, the required sample size is 26 patients per group (a total of 52 patients to be analyzed). With a lost-to-follow-up rate of 10%, 29 patients per group (a total of 58 patients) are required.

#### Populations to be analyzed

The intention-to-treat (ITT) analysis set consists of all patients entering the study (i.e. all patients who are randomized and receive a patient identification number) and receiving at least one treatment. According to the ITT principle, all patients are analyzed as belonging to their randomized treatment, regardless of whether treatment is refused or discontinued, or whether other protocol deviations are known.

The per protocol (PP) analysis set consists of the ITT analysis set with no major protocol violations. Major and minor protocol deviations will be identified by medically trained staff before database lock.

The safety population consists of all patients receiving at least one treatment and who undergo at least one post-baseline safety assessment. The statement that a patient had no adverse events also constitutes a safety assessment.

#### Statistical analysis

The cumulative number of AK lesions (sum of all AK lesions from visit 2 to visit 6) is defined as the primary endpoint. A point estimate is presented as mean and standard deviation accompanied by the corresponding 95% confidence interval. Furthermore, the primary endpoint is compared between the MAL/daylight-PDT and the cryosurgery group by means of an analysis of covariance (ANCOVA) with treatment (MAL/daylight-PDT and cryosurgery) as fixed factor and initial AK at baseline and center as covariates. The results are presented in terms of least-squares means and associated 95% confidence intervals. All efficacy analyses are carried out on the intention-to-treat (ITT) population and will be two-sided at the significance level of 0.05.

All secondary endpoints are analyzed in a purely exploratory manner and are summarized by means of descriptive statistics. Thus, *p*-values and corresponding confidence intervals are only descriptive in nature. Safety variables are analyzed descriptively. Adverse events are presented in frequency tables. In addition, adverse events are tabulated by severity and relationship to study treatment.

### Quality assurance

Study monitoring is conducted by experienced monitors. Monitoring is based on an a priori developed monitoring plan that includes inspecting the completeness of documentation and comparing the documentation with the source data.

Source data verification (SDV) is carried out at regular intervals. In all patients, the following data are compared with the source data: Basic patient documentation (sex and date of birth), inclusion and exclusion criteria, localization of AK study lesions, dates of all study visits, primary endpoint, adverse events, and the reason for premature study termination, if applicable.

### Data management and archiving

The patient identification list is controlled by the Investigator/Principal Investigator and will not be forwarded to other parties. Data recorded by pCRF will be pseudonymized. The responsible data manager monitors compliance to data security and protection rules according to Standard Operating Procedures (SOPs) and Working Instructions (WI).

All data management activities are conducted within a consistent, auditable, and integrated electronical environment (data security, data entry, and data validation). The responsible data manager takes care that no unauthorized access to the computer system takes place. By conducting adequate data backup procedures, the data manager makes sure that no data will be lost. Only specified persons involved in the trial and sworn to secrecy get authorization to enter or access the data trail of all transactions. Range, validity, and consistency checks are implemented into the system for application during data entry. After the first verification of the CRFs, all data collected on the CRFs are entered into the study database. Data entries as well as data modifications and data corrections are routinely recorded via the audit trail. In the case of necessary corrections or existing data inconsistencies, data queries are consecutively generated by the data manager. Queries are sent to the Principal Investigator/Investigator. Afterwards, data in the database are corrected according to the answered and resolved queries. After the database lock, data are imported into standard statistical software systems and forwarded to the biometrician in charge.

Archiving of the clinical database including the audit trail is conducted by the responsible data manager in a machine-independent format. The originals of all essential trial documents are filed in the TMF that will be stored at the archive of the Center for Clinical Studies Regensburg (ZKS) for at least 15 years after preparation of the final report. The investigators files all site-specific documents (e.g. correspondence with ethic committee), the patient identification list, the signed informed consent forms, copies of the pCRFs, and general trial documents (e.g. protocol, amendments) in the ISF, which will be also archived for at least 15 years at the individual trial sites.

### Ethical and regulatory aspects

The clinical study will be conducted in accordance with the German Drug Law (Arzneimittelgesetz, AMG), national and international GCP guidelines, and the Declaration of Helsinki, each in the applicable version.

In agreement with the German Drug Law (AMG) and ‘its adjunct GCP regulation (GCP-V)’, the study protocol was submitted to and approved by the Federal Institute for Drugs and Medical Devices (*Bundesinstitut für Arzneimittel und Medizinprodukte,* BfArM). Furthermore, the responsible regulatory authorities were notified about the study before patient recruitment could commence.

This clinical study protocol was submitted to and approved by the Independent Ethics Committee (IEC) of the Medical Faculty of the University of Regensburg, Germany.

## Discussion

The efficacy of daylight-PDT for the treatment of AK has been proven in several controlled studies [11, 13, 14]. A recently published phase III study conducted in Australia showed that DL-PDT with MAL for mild AK is as convenient, similarly effective, and nearly painless as conventional PDT [12]. Complete remission of AK at week 12 after daylight-PDT was 89% and 93% after conventional PDT. Pain was significantly lower during daylight-PDT (VAS score 0.8 vs. 5.7). The most recently published phase III study conducted in Europe also showed that DL-PDT is as effective as conventional PDT with methyl aminolevulinate cream, but better tolerated and nearly painless, and has high patient-satisfaction scores in patients with mild or moderate AK on the face or scalp [11]. Thus, DL-PDT with MAL is an effective, almost painless, and patient-friendly treatment for patients with AK that provides excellent cosmetic outcome. DL-PDT requires little equipment, reduces the physician’s time in the practice, and provides high patient satisfaction and motivation for retreatment.

Since field cancerization is considered a chronic skin disease and new AK lesions tend to develop even after effective therapy, preventive concepts are highly desirable. So far, no studies are available on daylight-PDT for the prevention of AK. In immunocompromised patients, conventional PDT has been shown to have the potential of delaying or reducing the development of new AK lesions when used in larger body areas. Investigations in the context of preventing non-melanoma skin cancer in renal transplant patients have shown that MAL-PDT delays the development of new lesions after one single field-directed treatment [18]. Furthermore, the potential of repeated long-term MAL-PDT (PDT every 6 months) in renal transplant recipients is being investigated over a period of 5 years. An interim analysis at the 3-year follow-up has shown the significantly delayed onset of AK as compared to untreated skin. The cumulative number of AK lesions was *n* = 8 in PDT-treated skin versus *n* = 43 in untreated skin [19]. In another study, 81 organ transplant recipients with AK lesions on different body sites were treated with two cycles of MAL-PDT as compared to cryosurgery [20]. Significantly less new AK lesions were present 3 months after PDT. After 27 months, the difference was not significant any more, suggesting that repeated treatments may be necessary for a prolonged preventive effect. ALA-PDT with blue light conducted every 4 to 8 weeks for 24 months reduced the development of new lesions after the study period by 95%. Only one study investigated the preventive potential of field-directed PDT in immunocompetent patients with cancerization on the face and scalp. In that study, ALA-PDT showed a significant delay of about 6 months until the development of new AK lesions as compared to the control sites [21].

Chronically sun-damaged skin is also characterized by sallowness, teleangiectasias, hyperpigmentations, roughness, and wrinkles. Therefore, simultaneous treatment of AK and photodamage is highly desirable. Several studies have reported on the rejuvenating effects of PDT [22–24], such as reduced fine wrinkles, mottled hyperpigmentation, tactile roughness, skin texture, teleangiectasias, facial erythema, and sallowness [25]. Only coarse wrinkles and sebaceous gland hyperplasia were not significantly improved. The cosmetic effects of topical PDT are supported by immunohistochemical analysis that showed both upregulation of collagen production and increased epidermal proliferation [26, 27]. Several studies have also shown a reduction in elastotic material in the dermis [28, 29]. These molecular effects together with the disappearance of Tp53, a marker for epidermal carcinogenesis, may explain why PDT is able to reverse the signs of photoaging [30].

Based on the findings after conventional PDT, the innovative research issue addressed in the present study is to investigate the prophylactic effect of repetitive PDT on the development of AK. As a secondary study endpoint, the rejuvenating effects of repetitive DL-PDT will be examined for the first time.

The reference therapy used in this clinical trial is cryosurgery − a frequently used treatment modality for AK that is recommended as a standard therapy in national and international guidelines and was used in most studies as standard therapy comparator for PDT. Complete remission rates are between 67 and 97% [31, 32]. Our aim is to compare a lesion directed standard therapy (cryotherapy) with a field directed therapy (DL-PDT) with respect to an expected prophylactic effect of a repetitive field directed PDT.

The results of this clinical trial may be of practical relevance for dermatologists and the numerous patients with chronically sun-damaged skin and AK, who have a high risk of developing new AK lesions that may progress into invasive squamous cell carcinoma. The demographic development will further increase the number of patients affected. So far, only a few treatment options for preventing AK are available, for instance sun avoidance and the consequent application of sunscreen, DNA repair enzymes, and oral nicotinamide [33, 34]. If the results of this study prove the efficacy of this treatment option, the preventive use of repetitive DL-PDT may be of great value for many patients because of the lack of other prophylactic treatment strategies.

Other clinically relevant questions may arise, such as DL-PDT as the possibly optimal treatment for immunocompromised patients or the role of DL-PDT as a preventive therapy for reducing the incidence of squamous cell carcinoma. These questions cannot be answered by the present trial and would require more specific protocols.

This clinical trial will assess the effectiveness of repetitive DL-PDT in treating and preventing AK in immunocompetent patients with sun-damaged facial skin. Patients of the control group will receive cryotherapy. This trial will also investigate the potential rejuvenating effects of repetitive DL-PDT.

## Abbreviations

AE: adverse events
AK: actinic keratoses
ALA: aminolevulinic acid
AMG: German Drug Law
ANCOVA: analysis of covariance
BfArM: Federal Institute for Drugs and Medical Devices
DL-PDT: Daylight-PDT /Photodynamic therapy with daylight
ITT: intention-to-treat
MAL: methyl aminolevulinate
PDT: photodynamic therapy
PP: per protocol
SAE: serious adverse events
SDV: Source data verification
SOPs: Standard Operating Procedures
VAS: visual analogue scale
WI: Working Instructions
